# Between Ecological Psychology and Enactivism: Is There Resonance?

**DOI:** 10.3389/fpsyg.2020.01147

**Published:** 2020-06-03

**Authors:** Kevin J. Ryan, Shaun Gallagher

**Affiliations:** ^1^Department of Philosophy, University of Nebraska at Omaha, Omaha, NE, United States; ^2^Department of Philosophy, University of Memphis, Memphis, TN, United States; ^3^Faculty of Law, Humanities and the Arts, University of Wollongong, Wollongong, NSW, Australia

**Keywords:** resonance, enactivism, ecological psychology, jazz performance, improvisation

## Abstract

Ecological psychologists and enactivists agree that the best explanation for a large share of cognition is non-representational in kind. In both ecological psychology and enactivist philosophy, then, the task is to offer an *explanans* that does not rely on representations. Different theorists within these camps have contrasting notions of what the best kind of non-representational explanation will look like, yet they agree on one central point: instead of focusing solely on factors interior to an agent, an important aspect of cognition is found in the link or coupling between an agent and the external world. This link is fluid, dynamic, and active in a variety of ways, and we do not need to add any internal extra something in the perception-action-cognition process. At the same time, even devout defenders of ecological psychology and enactivism recognize that plenty happens inside an agent during cognition. In particular, no one denies that the brain plays an important role. What, then, is the role of the brain if it’s not in the game of representing the environment? One possible option is to describe the brain as a resonant organ instead of a representational organ. In this paper we consider the history of resonance in more detail. Particular focus will be placed on two different sets of approaches that have developed the concept of resonance: a representational reading of resonance and a non-representational, dynamic account of resonance. We then apply these accounts to a case study on music performance, specifically in the context of standard tonal jazz. From this application, we propose that a non-representational resonance account consistent with both enactivism and ecological psychology is a viable way of explaining jazz performance. We conclude with future considerations on research regarding the brain as a resonant organ.

## Introduction

Orthodox ecological psychologists and enactivists agree that the best explanation for a large share of cognition is non-representational in kind. Such antirepresentational sentiments are noted explicitly by Claire Michaels and Zsolt Palatinus in their 9th commandment of ecological psychology: “thou shalt not make unto thee any mental image or likeness *of anything*” (2011, p. 25, emphasis added). In the enactivist church, Hutto and Myin (2017) are among the high-priests who declare it a mortal offense to espouse representations (also see Varela et al., 1991; Gallagher, 2017). On such views, mental representations are abstractions that do no real explanatory work. In both ecological psychology and enactivist philosophy, then, the task is to offer an *explanans* that does not rely on representations.

Different theorists within these camps have contrasting notions of what the best kind of non-representational explanation will look like. They nevertheless all agree on one central point: instead of focusing solely on factors interior to an agent, a good part of cognition is to be found in the link or coupling *between* an agent and the external world. This link is fluid, dynamic, and active in a variety of ways. Thus, the well-situated agent will pick up invariant information from the environment or engage with its world – either by choice (in the case of self-driven actions) or attraction (when something from the environment solicits and/or grabs the agent’s attention) – which will in turn drive iterant loops of perception, action, and cognition. One likewise does not need to add an internal extra something in the perception-action-cognition process.

At the same time, even devout defenders of ecological psychology and enactivism recognize that plenty happens inside an agent during cognition. Furthermore, although a full, embodied story will require taking into account many bodily and affective aspects of cognition (Colombetti, 2014), including even activity in the gut guiding behavior both in concert and independently from brain activity (Davidson et al., 2018; Liang et al., 2018), no one denies that the brain plays an important role. What, then, is the role of the brain if it’s not in the game of representing the environment?

One possible option is to describe the brain as a resonant organ instead of a representational organ. In what follows, we begin (in Section “RESONANCE: METAPHOR, MECHANISM, OR SOMETHING ELSE?”) by considering some of the history of resonance, especially in contact with the work of James J. Gibson, in more detail. Particular focus will be placed on two different sets of approaches that have developed the concept of resonance: (1) a representational reading of resonance and (2) a non-representational, dynamic account of resonance. Section “RESONANCE IN MUSIC PERFORMANCE” then applies these accounts to a case study on music performance, specifically in standard tonal jazz improvisation. We then conclude the paper with future considerations on research regarding the brain as a resonant organ.

## Resonance: Metaphor, Mechanism, or Something Else?

While the concept of resonance has played a role in the ecological psychology literature for over 50 years, it remains theoretically underdeveloped (cf. Raja, 2018, 2019). The idea that the brain resonates with the world instead of representing it first appeared in James J. Gibson’s work on perception and the senses. In Gibson’s words (1966):

Instead of supposing that the brain constructs or computes the objective information from a kaleidoscopic inflow of sensations, we may suppose the orienting of the organs of perception is governed by the brain so that the whole system of input and output resonates to the external information. (p. 5).

To understand the notion of resonance, following Shepard (1984), we can consider the case of how a piano resonates with external sounds as a prototype example of a resonant system. Assume you have a guitar and are standing near a piano. You pluck an open C-string and the piano strings will shortly start vibrating in resonance with the soundwaves in the air. But the piano’s C-strings will not be the only ones that vibrate in response to the original guitar note. Other strings with various harmonic relationships to C will, at the same time, become excited and start to vibrate as well, defined by specific constraints: “Resonators respond differently to the same stimuli, depending on their tuning” (Shepard, 1984, 433). There will also be amplification and resonance of the strings happening as a result of being inside an instrument. Indeed, there can be a variety of “modes of resonance” that occur in response to complex stimuli, such as an entire chord being strummed instead of an individual note (a point further discussed in Section “Representational Resonance”). We will return to this example in various parts of this paper.

While the relationship between resonance and instruments is clear, one may wonder if there is any coherent way to make sense of the idea that brains and neurons resonate in similar ways to musical instruments. Neurons, after all, are not strings or hollow tubes; they don’t literally vibrate. They do however come to be involved in patterns of oscillation, firing in dynamical connection with other neurons or groups of neurons. Varela (1996), for example, had proposed a role for transient spatiotemporal patterns of synchronous neural activity in explaining cognitive events.

For example, one resonant assembly could transiently bind together the different populations of neurons involved in analyzing the shape, color, and motion of a visual object, and this temporary assembly would constitute a neural substrate for the transient perception of a visual object. (Cosmelli et al., 2007, 737)

In their review article “Resonance, oscillation and the intrinsic frequency preferences of neurons,” Hutcheon and Yarom, 2000 explore the link between different brain rhythms and different perceptual and/or behavioral states. In their words, “A series of firmly established empirical associations with the behavioral states of organisms provides compelling evidence that brain rhythms reflect basic modes of dynamical organization in the brain” (2000, 216). Further developments in the field have helped establish that these resonance processes, especially insofar as they are phylogenetically preserved, may serve some functionally relevant role (Buzsaki and Draguhn, 2004). Similarly, the development of Adaptive Resonance Theory (ART) (Carpenter and Grossberg, 2010; Grossberg, 2013) has attempted to bring together these neuroscientific findings into a general cognitive theory^1^.

It is not clear that the concept of resonance in the brain is entirely captured by the notion of neural oscillations and rhythms, however. Sometimes the notion of resonance has been invoked in social cognition as two agents resonating with each other. One mechanism for this social sense of resonance is the mirror neuron system, which activates both when an agent performs an action, such as grasping a ball, and when that agent sees a conspecific performing the same action (Rizzolatti et al., 1996). Resonance within the brain in this case involves a neural system being activated by (or being sent into an oscillating pattern by) a bodily or environmental event. Other times resonance is even more metaphorical and is simply equivalent to general neural processing in response to the environment.

Considering these several distinct notions of resonance, the musical instrument example does not capture everything essential in the concept. Gibson, for example, further clarifies a possible passive misreading of resonance by noting that “The ‘resonating’ or ‘tuning’ of a system suggests the analogy of a radio receiver. This model is inadequate because there would have to be a little man to twiddle the knobs. A perceiver is a *self*-*tuning* system” (1966, p. 271, emphasis in original). The ability to self-tune is essential for making clear that, unlike passive systems or artifacts, cognitive resonance is not just something that the external world forces onto the organism. Indeed, sometimes resonance can occur without standard environmental inputs, such as during dreams or hallucinations. Such cases provide support to Gibson’s idea that there is an essential aspect of self-tuning involved in the system. Accordingly, embracing the idea of the brain as a resonant organ does not presuppose that the brain is a passive organ, since certain aspects of enactive resonance are not always themselves a passive process of information pick-up. Inhibitory processes can intervene, and the brain can activate in anticipation of some possible experience or activity.

For our immediate purposes, besides establishing a role for neurons resonating with each other, self-tuning may be best understood along with the idea that the brain is never isolated from the body or environment, and it always operates within this larger system to some degree. Accordingly, past experiences can set up the parameters of the resonance processes that will shape ongoing experience of the environment, which may, in turn, re-attune resonance processes. In cases of perception and action, the connection between organism and world will be a direct dynamical coupling. In cases of imagination or hallucination, the connection may not be one of direct coupling, but the patterns at play will still, to some degree, bear the mark (be similar to, or reactivate) some of the previous connections between agent and environment that correlated with perception and action in the first place (see, e.g., Lotze et al., 1999; Lee et al., 2012).

The move to highlight a self-tuning aspect to resonance alone does not solve a related issue about the nature of agency in resonance. Put succinctly, if the central claim of resonance is that all the brain is doing is resonating with ecological information, then it is not clear why any additional processing should occur inside the organism. Yet some of the choices made by an agent extend beyond information available in the environment. There is thus, it seems, a need for internal processing that cannot be explained solely by appeal to resonance. If we were working on the idea that resonance is a metaphor, then this result might be rather benign. However, all of the main theorists we will be discussing – Vicente Raja, Thomas Fuchs, and Roger Shepard – agree that resonance is a material process rather than a mere metaphor. As a result, in exploring their respective approaches, we need to ask what is the best way to understand this relevant additional internal processing?

To achieve this goal, the remainder of this section is broken into two subsections. The first considers a representational reading of resonance. The second considers a non-representational alternative, which we shall refer to interchangeably as a dynamic reading of resonance. Since these sections will serve as the introduction to various takes on resonance, most of the critical work that has been leveled against them will be reserved for later sections of the paper.

### Representational Resonance

A main and early proponent of the representational account of resonance is Shepard (1981, 1982, 1984). Some of his ideas on the topic were briefly introduced in the above case of the piano resonating with the guitar. A few more need to be added to give a complete sense of his account. In particular we need to consider the notion of a complex nesting of resonance within a cognitive hierarchy and the notion of complementarity instead of isomorphism between resonating systems (Shepard, 1984).

Since we have moved from mere metaphor to a material process, we likewise need to consider how resonance may be physically realized in a particular system such as the brain. Shepard (1984, 433) flags three upshots of resonant systems that will have implications for the use of this concept in cognitive science. First, resonant systems will have constraints that are shaped both by what is being tuned and how it is tuned. Second, there are multiple ways to excite a resonant system. Third, and in relation to the second point, there are different modes of excitation in a resonant system.

We can see these three elements in play by returning to the case of the piano as a resonant system (Shepard, 1984, 433-4; also see Raja, 2019 for different musical instrument examples). In regard to the first point, the harmonics that a piano resonates at will be impacted by how the strings have been tuned. The resulting sounds will simultaneously have a particular timbre that is shaped by various physical features of the piano, in contrast to the physical characteristics of a guitar, sax, flute, drums, etc. A similar relationship holds in the case of neurons, where some elements of their resonance patterns will be shaped by how they have been “tuned” over a lifetime of engaging with the environment, while others – such as theta, beta, or gamma brainwaves – are inherent to their nature and place in brain architecture.

In regard to the second point, the activation of strings can occur from various different signals and sources, including but not limited to soundwaves that are strong and identical to a given tuning. For instance, in addition to matching tones, resonance could be realized by sounds that are harmonically related, sources from incomplete tones, or sounds with variable energy and force. Finally, for the third point, activation of a piano is not achieved only by the strings resonating with external sounds. It may also be activated by playing other notes or chords on the piano itself, as well as plucking or striking the string directly by hand.

There are several limitations with a piano as our guiding example, in addition to those canvased above, namely the piano is not able to be self-tuning nor begin playing on its own accord. Not even a sophisticated pianola would entirely solve these problems. As such, we now turn to address this point through the role of a complex agential hierarchy in place for an organism but not for an inanimate object.

Shepard (1984) notes that it is important to conceptualize the various resonant modes as organized hierarchically within the system. When combined with endogenous and exogenous sources of excitation, this hierarchical system is able to pick out or represent the complex web of perceptual invariants in the environment. Sometimes it does so by moving up or down levels, perhaps focusing on high-level, general kinds (e.g., one hierarchical organization represents the sound of a doorbell) or low-level, more specific features (e.g., a lower-level part of that hierarchy represents a specific pitch regardless of timbre, or perhaps another hierarchical organization represents my parent’s doorbell instead of doorbells in general). Other times a higher level may resonate directly without requiring the excitation of lower levels of the system, such as if we are simply thinking about or imagining a doorbell ringing. As a result, Shepard suggests that these internally and externally driven sources of resonance are consistent with the idea that perception is “externally guided hallucination,” a claim that has since become part of predictive processing accounts of perception and action (Clark, 2013, 2016).

This view of perception as “externally guided hallucination” presupposes a view of the brain as more than just mirroring the external world. Shepard here suggests that some aspects of the brain are not directly resonating with the outside world, especially in cases of non-ideal perceptual environments. Instead of direct perception, the brain is “sympathetically excited” by purely internal activities (1984, 436). Understanding perception as a subset of hallucination, heavily subsidized by internal processes of imagination, is a view rejected by the dynamical approaches to resonance that we consider in the next section.

Shepard’s account furthermore puts pressure on the role of resonance as a kind of isomorphic mirroring between agent and environment. He suggests that it is better to think of the resonance between brain and world as a case of *complementarity* patterns. For instance, consider one of Shepard’s favorite examples of a key and a corresponding lock. There is a direct correspondence between a key and the lock and it is a necessary condition for the proper functioning of the locking system. However, we would be hard pressed to say that the key is isomorphic to the lock. Instead of matching, this relation between key and lock is complementary; the key complements the lock in the right sort of way to unlock or lock the door. We will return to Shepard’s account again through the work of Charles Nussbaum in considering musical performance below and the idea of complimentary will play a central role in that context as well.

One may be tempted to claim here that moving away from isomorphism may itself be a move away from a representational account. Complementarity, however, may still characterize representational function insofar as it meets what Ramsey (2007) has called “the job description challenge,” where the mark of representational processes is that they serve an explicit representational function. Consider the case of perception in ambiguous or non-ideal perceptual situations. In these cases, it is important that the brain can complement the available information with its own productions rather than simply pick up information and react. Insofar as some self-tuning patterns of resonance can be stimulated and maintained by various parts of the brain, they can stand in for missing aspects of external stimuli, supplementing when the stimulus is too impoverished. This stand-in may occur in a manner that addresses the job description challenge.

Shepard (1975) himself explicitly notes the representational components of his theory in several papers (1975, 1984). The sorts of representations he has in mind here are often centered on mental imagery. Thus, in his words, “I conjecture that Gibson disavowed the term *mental image* because he could not imagine what sort of thing a mental image could be…However, in neglecting the representation of objects and events that are not physically present, Gibson seems to have given up too much” (1984, 420, emphasis in original). Shepard further cites evidence for similar durations for mental image rotation across perception and imagination to suggest that an agent must be working with a representation of an external object during imagination, when the object isn’t actually present.

This notion of mental imagery in Shepard can further be thought of as representational insofar as it lines up with recent notions of structural representations. Following Piccinini (2018), this kind of representation includes (2018, 3):

(1) A homomorphism (partial isomorphism) between a system of internal states and their target,
(2) A causal connection from the target to the internal states,
(3) The possibility for the internal states to be decoupled from their target, and
(4) A role in action control.

All four of these features are indicative of Shepard’s account. In addition, following Ramsey (2007), Chap (6), it seems that structural representations at least *prima facie* qualify as satisfying the job description challenge.^2^

### Dynamic Resonance

We will now introduce two alternative approaches to resonance from enactivist and ecological psychology backgrounds. When looking at points of similarity between these two non-representationalist camps, we shall refer to them collectively as constituting a dynamic notion of resonance.^3^

Thomas Fuchs, like Shepard and Gibson, highlights the fact that the notion of resonance comes from considerations about acoustics and oscillations. He further draws out the acoustic language to a different metaphor of the brain as taking part in jazz improvisation. In Fuchs’ words, “the brain is not the conductor of the body; rather, it is like a musician in a group of jazz musicians jointly improvising on the basis of certain chords” (2018, p. 134). This is similar to Gibson (1979/2014) motto “behavior is regular without being regulated” (1979/2014, p. 215)^4^. This improvising jazz picture can be contrasted to traditional cognitivist assumptions that neatly partition sensory input/brain processes/action output and treat the brain as a conductor. For cognitivists, the brain may be part of the body (as a conductor is part of the orchestra) yet it is essentially distinct from more strictly embodied activities and, instead, plays its main role in guiding our actions in response to our sensations (reflected in an understanding of the conductor somehow playing the orchestra as their “instrument”).

In contrast to cognitivism, a dynamical account takes as an important insight that, while the brain plays some essential and likely unique role in cognition, (1) it is not in the business of controlling the entire process of cognition, (2) it is necessarily and inextricably responsive to various aspects of the overall cognitive system in deep and consistent ways, and (3) it cannot be understood in isolation from other processes happening across the body and environment. Even the soloist in a jazz band is similarly bound by these sorts of constraints, assuming that the band is structured more around improvisation and less around playing composed music.

Fuchs suggests that the acoustical focus likewise brings the essentially temporal nature of cognition to the fore. As a result, in his words, “Resonance contains a dynamical as well as a rhythmical element and thus establishes a *temporally* overarching relation between the systems involved…’resonandum’ and ‘resonans’ thus cannot be separated” (2018, p. 166). Such inseparability is furthermore taken as a sign that the explanation of resonance, and of the brain in general, cannot be representational in nature; a representational account, if nothing else, must at least allow for some form of decouplability between the initial representational vehicle and its representational content (Gallagher, 2017, Chap. 5). In contrast, the *relata* in resonance in some way need to remain coupled for resonance to work: brain-body, organism-environment, you-me, etc.

Resonance can be found in two intersections, according to Fuchs. First, the brain and body resonate with each other in a dynamical, intertwined, circular process that involves homeostasis. Damasio (2010, 21) calls this a ‘resonant loop’. This brain-body resonance is tied into the fact that the brain is “*the ‘integral’ of the overarching process of life* which encompasses the whole organism” (Fuchs, 2018, 119, emphasis in original). From the level of densely interconnected brain activity across the brainstem and cortex, to the role of affect as essential to cognitive activity, and out further still to the densely intertwined efferent and afferent feedback between the brain and non-neural body, changes in one locus will reverberate and resonate with all other areas in the system.

Second, there is a resonance between an organism and the environment. This particular resonance occurs through a “dynamic set of isomorphic patterns” that develop between the brain, body, and world. An example of isomorphic resonant patterns would be when a specific (or similar) brain pattern occurs in response to the presence of a specific (or similar) environmental context. Moreover, neural evidence has shown that brain patterns change in response to learning new habits and skills, such as the increase in musical ability resulting in different neural activations compared to novices first learning how to play (Oechslin et al., 2013). Such results suggest that the dynamics of these isomorphic patterns are skill dependent on experience instead of arriving hardwired ahead of time. Fuchs’ emphasis on the isomorphic quality of resonance is tied to his endorsement of an Aristotelian formulation of intentionality, where the mind takes on the form (*eidos* or *morphe*) of the perceived object (Fuchs, 2018, 166ff).

If resonance occurs across brain, body and environment, however, then distinguishing two sorts of resonance is not enough to provide the complete story. While multiple types and scales of resonance will be essential to understanding what it means for the brain to be a resonant organ, these can be further parsed out to give a more detailed sense of exactly how all of them are ordered. According to Vicente Raja (2018, 33), there are three possible target scales for resonance: (1) the agent-CNS (Central Nervous System) interaction, i.e., “the CNS activity in relation with the overall activity of the agent in her environment,” (2) body- or inner-CNS interaction, and (3) CNS-environment interaction. While Fuchs considers resonance across these scales, he does so without marking them in these terms. Keeping them together is ultimately important for the overall account of dynamic resonance. Yet distinguishing them is equally important since it makes the contours of how different kinds of resonance relate to each other clearer than they would be otherwise.

The inner-CNS scale, which would narrowly track the first resonance presented by Fuchs, involves an important form of resonance between the CNS and other parts of the body, no doubt. The problem with placing a focus solely on this level to understand perception and/or action is that the inner-CNS scale fails to track variables at the ecological level, which are needed for the complete brain-body-world explanation of perception and action.

The CNS-environment scale, in contrast, fails to account for important ways that an agent is able to modulate and alter their interactions with the environment. More specifically, on this scale, the focus would move directly between activity of the CNS and the environment, without taking consideration of the peripheral nervous system or bodily affects. And even though the agent may not have complete control over their relationship to the environment, there is more endogenous processing going on outside of the CNS itself.

At the agent-CNS scale, which Raja takes to be the correct target for explanation, the focus is how activity of the CNS resonates with the organized activity of an agent within her environment. This scale necessarily requires drawing on the full suite of intra-organism resonances (e.g., intraneural resonance among different neurons and brain regions coupled with homeostatic resonance between the brain, heart, stomach, and lungs), including those under agential control and those outside of it, and the resonances between the embodied agent and her environment. This scale is also equivalent to a full integration of the two resonances described by Fuchs.

Raja further develops the notion of dynamic resonance by first appealing to Michael Anderson’s account of “neural reuse” for an account of resonance in the brain and, second, Dynamical Systems Theory for an account of ecological, i.e., organism-environment, resonance. According to Raja, these are compatible theories, with structural and theoretical parallels. Neural reuse allows for a rich flexibility and sensitivity in patterns of functional connectivity to the demands of different cognitive tasks, consistent with the notion that neural resonance may change as an adjustment to different goals and tasks. As Raja notes, the idea of neural reuse is precisely expressed by Gibson in an unpublished manuscript: “a given set of neurons is equipotential for various different functions in perception and behavior. The same neuron may be excited for different uses at different times. [Accordingly] neurons, nerves, and parts of the brain have a vicarious function. A nerve cell is not the same unit in a different combination of nerve cells” (cited in Reed, 1988, p. 224).

According to proponents of neural reuse, different conjuncts of brain areas will be dynamically (re)configured as functional units depending on the task, setting up a specialized resonance in response to particular cognitive demands. The additional dynamic coupling between the brain understood as a resonant organ founded on neural reuse, on one hand, and the environment, on the other, is defined in relation to a common ecological variable that constrains the actions of the agent. Accordingly, we are able to say that the intra-organism system (i.e., the agent) as one system *resonates* dynamically with the environment in order to engage with the world. For Raja, these two systems integrate, via resonance, to form one overarching dynamical system. While there are cases of linear coupling between different (sub)systems in cognition, the vast majority of cases involve non-linear coupling [a concept also embraced by Fuchs (2018, 223)], which would imply some constraints on any isomorphic resonance). Non-linear cases are marked by an interdependency between the two (or more) systems under consideration (Van Gelder, 1995). Such non-linear coupling may be read in line with a shift from understanding the cognitive system as an agent connected with the environment to, instead, focusing our cognitive explanation on the organism-environment as itself a single relational cognitive system. Indeed, such shifts are important for moving past the internalism/externalism dichotomy that plagues traditional accounts of cognition.

Raja postulates that the resonance between these two dynamical systems is one wherein the ecological scale constrains the intraorganism scales but not vice-versa. In his words, “*to explain resonance is to account for the coupling of the dynamic systems at the ecological and intra-organismic scales in terms of the ecological variable that constrains a given agent-environment interaction*” (2018, 41, emphasis in original). The importance of this directionality comes from a core commitment of ecological psychology to the idea that the environment will play a particularly strong guiding force in organism-environment interactions. Moreover, Raja suggests that considerations of both biological and explanatory plausibility push toward an unequal relationship in favor of ecological constraints on the organism over and above any organism constraints on the environment.

At the same time, Raja has noted that information at the ecological scale is ultimately developed in the interplay of organism and environment (Raja, 2020; also see Raja and Anderson, 2019). Raja appeals to the work of William Warren on behavioral dynamics, among others, to help clarify this interactive process. According to Warren, the challenge of behavior is accounting for the required mix of stability and flexibility utilized by an agent when engaged with the world. Furthermore, in his words, “[f]rom the agent’s point of view, the task is *to exploit physical and informational constraints to stabilize the intended behavior*” (Warren, 2006, 359, emphasis in original). Such an exploitation is clearly an active process on the part of the agent, which entails that the ecological variable should be understood as including the interplay between organism and environment as part of the process, rather than operating as a mere external constraint.

A major point of similarity, then, between Fuchs and Raja, and more generally between enactivists and ecological psychologists, is the appeal to dynamical non-linear coupling between brain, body, and world. In the case of action and perception, we should consider the role of enabling constraints (Anderson, 2015), where neural activity is constrained by higher-order, organism-environment dynamics. This does not mean that brain dynamics are passive. Rather, not only does brain activity function within the proper constraints of organism-environment dynamics but organism-environment dynamics are also (at least partially) enabled by brain activity. Thus, “the brain supports on-going behavior, to anticipate forthcoming behavior…. [which] allows a healthy codetermination of action by the actor’s history and context together with the momentary contingencies that choose the behavior that is enacted” (Van Orden et al., 2012).^5^ For both enactivism and ecological approaches, once we start looking at resonance processes in the brain, we are immediately led to consider the larger system of brain-body-environment.

## Resonance in Music Performance

Thus far we have considered the debate around resonance on a rather abstract level. In this section, we turn to a particular case of what happens in the brains, bodies and environments of musicians during music performance. Although resonance (including both acoustic and neuronal resonance) is an important part of all different kinds of music performance, we shall primarily focus on standard tonal jazz performance. Doing so will help adjudicate between the various positions displayed above and, we believe, will ultimately side in favor of a dynamical account of resonance. As a theoretical model in its current state, we acknowledge that our account is open to empirical verification or falsification. We furthermore hope that it may serve to help guide future empirical work in various aspects of music performance and pedagogy, and developments in these areas will loop back to further help develop our theoretical account accordingly.

To begin, we will say a few words about standard tonal jazz performance. When it comes to playing a jazz standard or song, the format follows three main steps. First, the band begins by playing the “head,” which is a statement (or implication) of the main melodic line that demarcates the song. Second, the band moves into the solo section. While different subgenres of jazz embody different expectations and constraints on solo practices, the main constraint for the standard format is harmonic in nature: the chord changes introduced in the head are kept consistent throughout the performance, and “correct” notes are dictated by following this chord progression. Melodic and rhythmic choices, in contrast, are largely left to the decision of the performer. Third, after everyone has taken one or two choruses to play their respective solos, the band plays the outro, which is often a restatement of the head, and the song ends.

During an improvised performance, resonance happens on several different levels.

• First, the individual performer resonates with the music. This is a resonance between the sounds one creates and the sounds in the environment (e.g., the sounds made by other musicians). Much of this resonance will happen at the moment of sound creation. It may be driven by (1) consciously anticipated,^6^ and sometimes planned, notes and/or (2) feedback from awareness of the sounds that are actually created during performance. On one hand, as the music unfolds, the performance environment is constituted as a niche of musical affordances. The sounds that a musician produces could thus successfully or unsuccessfully resonate with the affordances in the environment. On the other hand, anticipatory processes and any short-term planning involved while playing suggest intra-organism resonant loops constantly underlying the performance. The combination between these respective elements constitutes what Christensen et al. (2016) call a “mesh” between anticipatory control, practiced/skilled bodily movements, and the affordances presented by the music (see Christensen and Sutton, 2019; Gallagher, in press).
• Second, there needs to be an intersubjective and affective resonance between an individual’s performance and the performance of other musicians. This may be mediated by the music itself, by conscious, non-conscious, and non-verbal perceptual cues, or sometimes by verbal feedback during performance (see Høffding, 2019; Høffding and Satne, 2019).
• Third, in some cases there may also be resonance between the musical group and the audience. Depending on the performance context and individual musicians, this final resonance may be as interactive and important as the earlier kinds, act as a unidirectional constraint (e.g., the band is shaping the audience response but the musicians have little response to audience feedback), or rather unimportant to the unfolding of the performance.

Such resonances may or may not be understood as metaphorical in nature (non-metaphorical resonance may include neural resonance; see Large (2010) for a general overview of neural resonance in music). Either way, we decided on a heavily improvised performance practice as our case study since it presents a distinct explanatory problem for resonance in the form of a specific type of uncertainty. In musical performances that have preexistent, thick song structures, a large part of the cognitive work can be explained by appeal to a more stable performance environment (through, e.g., the use of a score or by the performance of a well-practiced song). This stable environment may involve either strong standing mental images of a music performance, a la Shepard, or it may provide a clear environmental variable that constrains intra-organismic resonance underlying action, a la Raja. Parts of these explanations may be carried directly over into jazz performance with little modification. Yet there is nevertheless an important difference between improvised and composed music to the extent that the actions of the musician are open to more immediate changes and on the fly decisions about the music. We will call this situation one of increased environmental uncertainty during jazz performance.

Uncertainty here doesn’t mean that jazz performance takes place in a poorly structured performance environment. Instead, it is meant to highlight the fact that, in addition to the importance of an agentive self-tuning of resonance, the performance space and the music itself do not impose any overly strong constraint on choices made during performance, even as they impose some constraints. Since these constraints are extremely flexible, there may be a worry that the invariant structures in the environment – a core aspect of ecological explanations – are not strong enough to stand on their own without additional internal processing of a kind eschewed by Michaels and Palatinus (2011). A soloist is not just a coupled oscillator resonating with patterns of their environment, after all. They also must create improvised choices in the moment of performance. To explain the creative possibilities of jazz performance, it seems that we must go beyond matching or strict isomorphic resonance to something more in either a representational or dynamic account of resonance.

In what follows, we will not be attempting to offer a complete account of how resonance operates during jazz performance. We will also not be offering an argument to show that a representational account of resonance cannot function to explain jazz performance. We instead will consider how the two different accounts canvased in the previous section – representational and dynamic – each make sense of jazz performance. Furthermore, we will also consider the limitations of a purely isomorphic account of resonance before we consider how more details about dynamically formed constraints between agent and environment can answer the main concern about how dynamic accounts can deal with the environmental uncertainty at hand.

### Isomorphic Resonance

While we suggested above that isomorphism is not the way to go, we grant that one of the simplest routes to respond to the challenge is to insist that isomorphism is all we need to explain musical performance. Because parsimony is an important part of scientific theorizing, we will begin with what seems to be the most parsimonious account of the jazz musician isomorphically resonating with external components of the environment in order to drive a performance forward. Indeed, after invoking considerations of parsimony in particular, one may wonder why we need more than isomorphic matching, at least in the case of resonance between an agent and the environment, or specifically with the music.

A purely isomorphic analysis runs into problems with its focus on matching resonant patterns in cases where one-to-one mapping is either impossible or not preferable. This may be an artifact of focusing on perception in non-improvisational settings, where one might claim a clear connection between the invariant structures of the environment and the actions of an organism. The worry may also be the result of the artificial limitations of incorrectly focusing on the CNS-environment scale (an isomorphism between brain and environment) instead of on the agent-CNS scale (brain-body-environment).

One possible response to this concern is that the neural patterns are only partially isomorphic patterns relevant for action, rather than abstract or ideal isomorphic patterns. After all, the patterns involved in resonance involve a rich affordance landscape, laden as it is with a variety of meanings for the organism. Thus, one may suggest that isomorphic resonance is not about locking down static environmental features but, instead, grasping patterns in the world through perception, or enacting patterns in the world through action, that ultimately allow agents to act on the rich, complex, and dynamic environment.

Unfortunately, while not entirely inconsistent, some parts of Fuchs’ analysis of isomorphic resonance in perception go against this reading. For instance, after appealing to Herbert Dreyfus and Charles Taylor on an Aristotelian view of the mind, Fuchs (2018) claims:

the brain could be conceived as a matrix, which like the mind is able to ‘receive all forms,’ that is to say, to take them over in its own structure as neural patterns or potentials. In the actual perception ‘mind and object become one,’ corresponding to an encompassing resonant system state in which the *same pattern or form is activated in the brain as it is displayed by the object*.” (p. 167, emphasis added).

In Aristotelian terms, the form of an object can be distinguished from its matter, and it is the form (*morphe*) that is replicated in neural patterns during perception. However, accepting this Aristotelian idea runs into a problem concerning exactly what it means to be isomorphic. What is the exact isomorphism between the taste of a good wine and its correlated neural state? Is the structural similarity of the isomorphism to be taken at a first level of isomorphism (e.g., the brain resonates with the invariant features for the taste of this particular wine) or a higher-order (e.g., the brain resonates with the invariant features of the experience of tasting this wine)? If taken in the first level sense, what does it mean to resonate with the “taste” of the wine? Indeed, which specific properties are actually being resonated with in cases of perception, aesthetic or otherwise? Since Fuchs account is supplemented by a theory of action and gestalt completion, he may be able to provide direct answers to such questions (e.g., the brain is resonating with the invariant features of the wine as it hits the taste buds^7^). Someone who only focuses on isomorphic resonance, in contrast, would be unable to provide this more detailed account needed to explain jazz performance.

This series of questions alone is not a reason to turn toward a representational account of resonance. However, it does suggest a need for having a more detailed account of resonance beyond isomorphism. In a similar manner, since the representational resonance account has a detailed way of answering these points, we will begin with it before turning to a dynamical account.

### A Need for Representations?

Facing an uncertain environment is often a main motivation for positing representational accounts in the first place. In the case of music perception, drawing on considerations from both ecological psychology and Roger Shepard, we suggest that Nussbaum (2007) account is one of the best developed of what could be construed as representational resonance applied to the case of music^8^. Before delving into the particulars of this account, however, there is one immediate issue about his clearly stated focus on an account of Western art music and its listeners (2007, 38-40) that needs to be addressed.

The worry here is that this account cannot be applied to jazz ensemble performance without some serious modifications. We grant that there is additional work, especially empirical in nature, that needs to be conducted before we can say that Nussbaum’s picture as given holds up well in the case of explaining jazz performance and the perception that goes on in jazz musicians during performance. Nevertheless, most of the basic tenets underlying his theory can be applied without much reworking, such as a particular role being placed on “acceptable” moves during the development of solos that respect certain tonal, chord, and key related constraints. While this focus on harmony does not fully exhaust an account for all important aspects of jazz performance, those extensions will be equally difficult for all accounts of resonance to satisfy, and thus we shall not consider them in more detail at the current moment.

Following Gibson’s definition of affordances, Nussbaum suggests that the physical music itself can act as an external representation for audience members and performers. It does so by being a series of invariant relationships, i.e., musical affordances, that are intertwined with motor and action responses to the music. If this kind of external representation were the only one in this account, it would be easily amenable to dynamical accounts of resonance. Even the most radical non-representationalist doesn’t claim that there are, strictly speaking, no representations in any parts of human cognition. Language, after all, is an essential part of different cognitive capacities and an example of a representational system *par excellence*.

External representations, however, are not the only sorts of representations for Nussbaum. He instead argues that musical surfaces are “a carrier or vehicle from which information can be extracted by performing appropriate transformational operations that are supported by *representations in the human mind-brain*” (2007, 23, emphasis added). These internal representations are musical rules implemented in the motor system, similar in kind to Chomsky’s (1965) rules in generative grammar and as developed by Lerdahl and Jackendoff (1983) in the context of musical meaning. More could be said about how this implementation process operates, but the details are unnecessary to motivate the main idea that internal representations are taken by Nussbaum as a necessary part of explaining brain function.

On this account, the role of representations seems to be even more important in improvised music because there is no score to act as an external representation storage for the music. Moreover, while the standard performance structure may set basic parameters within which a musician must perform, there remains a vast amount of possible decisions that a musician could make. Thelonious Monk, for instance, was well known for utilizing unconventional chord voicings during performance. His selection of such chords may have been available in the music, but it had to be interpreted and/or they had to be added to the piece in some way. Such decisions are indicative of a certain ambiguity for both how a jazz musician perceives the music and for how they decide what to produce next. They also mirror well the discussion of Shepard’s account from section Representational Resonance.

Speaking directly in regard to ambiguity in perception, Nussbaum echoes Shepard and notes that “degraded and ambiguous inputs immediately reveal the extent to which information extraction depends on pattern completion, which in turn requires internal representations and constructive procedures that operate over these representations” (2007, 34). Instead of isomorphism, the suggestion here would be that the better push is instead for complementarity between brain and world. Such complementarity, in turn, may require the brain to represent aspects of the world rather than to isomorphically resonate with them. Resonant isomorphism may beget either representationalism or non-representationalism; in contrast, non-isomorphism would appear to require some sort of complementary representationalism. The key question therefore becomes do enactivist and ecological accounts of resonance have the resources needed to explain the process of resonance underlying jazz performance without appealing to these kinds of complementary representations?

Based on these considerations, a representational resonance account of jazz performance may be sketched as follows: assume that a trumpet player is soloing during *My Funny Valentine* in a trio consisting of her, a drummer, and a piano player. From years of practice, the trumpet player has built up a rich store of internal representations regarding her playing possibilities, including an understanding of both rhythmic and tonal possibilities for performance according to idioms in the musical language of jazz. In this particular performance, she puts those representations to use in order to address the combination of both the song structure at hand as well as the performances of the rest of the group. The uncertainty and openness of the performance space – the particular chord phrasing of the piano or the subtle tempo shifts of the drums – are supplemented by internal representations, on this account, since the invariant features of the environment alone are not enough to secure a successful performance.

Considering this brief account and the turn to complementarity, the challenge for dynamical resonance can be restated as whether it can offer a story of complementarity without representations. Before turning to dynamic resonance, a preliminary point to consider here *contra* Nussbaum would be that his representational approach fails to acknowledge a possibility for metastability within the brain that provides the requisite flexibility, while not itself being a representational phenomenon. The brain can move into multiple different stable patterns of activation that are dependent on the specific dynamics of the action in play. Boxers, for example, will deploy different movements and fighting patterns depending on how close they are to their opponent (Rietveld et al., 2018). In a similar way, jazz musicians may choose to be more or less adventurous with their soloing based on factors such as trust, experience, and audience expectations. Such notions are not captured by the kinds of internal representations that Nussbaum utilizes in his account. While adventurousness is a broad concept, at the very least it seems to be captured by the musician’s self-selection regarding how much metastable behavior they engage in when listening and performing. This metastability in behavior is further likely to be supported by neural metastabilities such as those proposed by Friston (1997).

### Dynamical Resonance Returned

We currently have the following picture of dynamical resonance: dynamical resonance provides an explanation of how an organism picks up relevant environmental information, responds to it as needed, and acts without any sort of representing by the organism in any meaningful sense of the term. Enactivists maintain that agents enact a world – that is, they enact meaning - and this happens in a way that depends on a dynamical-relational coupling of organism/agent-environment. This enactivist view is fully consistent with an ecological interpretation of affordances as relational – i.e., that affordances are not agent-independent characteristics of the environment, but define an agent-environment relation. Resonance thus occurs in affordance-based responses to various aspects of the environment in order to support actions.

On this picture, the environment, in contrast to the agent, does not resonate with an organism. As described so far, the environment is there existing as a constraint or partial constraint. Understood this starkly, we are here faced with an issue: following Raja, if resonance only occurs when the musician is tightly constrained by an environmental variable, we run into a distinct problem with the uncertainty of the environment coupled with the flexible nature of improvised jazz performance.

This problem concerns the fact that even informationally rich and invariant features of a jazz performance still leave a wide latitude of possible choices for musicians. In other words, a musician may resonate with parts of the sonic world during a solo, yet their choices are not overly constrained by that resonance process. They may even refuse processes of resonance as much as become entrained by them.^9^ Part of Raja’s answer to this issue may be cashed out in regards to both neural reuse, which allows for a flexible engagement between an agent and their environment, and a role for behavioral dynamics (Warren, 2006). We suggest that this move is an essential part of responding to the worry. However, it leaves open whether we should consider the cognitive explanation in this case to be of different interrelated dynamical systems – the brain (where neural reuse happens), the musician and the environment – or one overarching dynamic system consisting of a single musician-environment system. For the sake of space, we shall primarily focus on the second reading here.

The latter interpretation of a single system runs into a *prima facie* concern based on the idea that an environmental variable must constrain the intraorganism scales. In short, the single cognitive system interpretation may go against Raja (2018) position that there’s a distinct environmental variable constraining the organism. To clarify what we take the heart of this worry to be, we are not concerned with the idea that the environmental constraint may be overly restrictive of an agent’s behavior, nor are we concerned that the focus on an ecological variable keeps us stuck on the ecological level without giving a proper explanation of the brain as resonant organ. Instead, the issue is how to make principled sense of an environmental variable if, as enactivists argue, cognition is fundamentally an enacted improvisation among the co-performing aspects of the brain, body, and world.

One response from ecological psychologists is to note that the environment is not separate from the organism but, instead, includes it (see Segundo-Ortin et al. (2019) for more information). Another plausible option is that a co-performance among these aspects doesn’t entail equal weight being distributed among all of them at all times. Enactivists need not rule out the idea that one factor may take the lead in certain circumstances. Resonance may moreover require that, in certain cases, it would make sense to talk about a variable ostensibly external to the organism or agent constraining the actions (or operating as boundary conditions on the dynamics) of the organism or agent. Instead of necessarily treating all aspects of the cognitive system as co-equals in the cognitive process, a proponent of ecological psychology may argue that there is reason for at least decomposing parts of the overall agent-environment system in ways where the balance of power, so to speak, itself can vary from circumstance to circumstance. Indeed, the appeal to enabling constraints mentioned above may be a way of grounding this sort of response.

Another important thing to note is that playing in a jazz performance is not identical to tasks such as walking down the street or moving furniture around the room.^10^ In enacting a performance, a musician is not only resonating with several different aspects of their environment, but, in addition, by playing the music they are creating important parts of their musical environment, capitalizing on musical affordances made by themselves and the other musicians. In this sense, the musical (as well as the intersubjective/social) environment is resonating with the performance. The self-tuning of the performer, or group, important in Gibson’s account of resonance, is at the same time a tuning of the environment. The other musicians in the group, for example, also resonate with the performance and with the music all of them are making together. Thus, we suggest that a jazz performance is one where there is mutual, looping resonances between the musicians, each other, and various aspects of their environment, important parts of which they are creating on the fly.

This mutual resonance points to what is missing from a mistakenly strong reading of environmental information acting as a unilateral constraint on human action. What is missing from it is an account of how humans and other organisms can be active forces that shape their environments. Since we can actively construct or reorganize an environment to enhance resonance processes, or to make the environment resonate with us, a full account of resonance must explain this part of the process as well, the importance of which is highlighted by theories of niche construction in particular (Laland et al., 2016). Such alterations of the environment can take place at quick timescales as well as over the course of an individual’s lifetime (or multiple lifetimes, if we are considering a species and not just individuals). While the theory of neural reuse from Raja’s account does reference some considerations about niche construction as it stands, it does not yet explain how organisms actively modulate their niches and the rest of the environment in real time.^11^ While making this shift does not necessarily require a radical rethinking of core tenants of ecological psychology, foregrounding a changing and sometimes ambiguous environment is important if we are to develop a full account of resonance moving forward, especially for musical performance.

As an example of this real time environmental modulation in jazz improvisation, consider how accompanying musicians act as affordances for a soloist. The choice a bassist makes between playing a walking bass line or a consistent pedal point will impact the affordances available to a soloing sax player and call for complimentary rather than isomorphic responses. At the same time, the soloist acts as an affordance for the other musicians, especially in the case of bebop and similar subgenres. The individual and collective choices of the musician(s) in such cases will have an immediate impact on the purportedly constraining environmental variable. This impact both gives at least a modicum of control to the performers to shape the song and provides a much more dynamic environmental variable (or set of variables) with which the musicians resonate during performance.

More could also be said here about the nature of action in jazz performance. Some cases or aspects of resonance may be isomorphic, as Fuchs suggests. Other cases or aspects of resonance will be non-isomorphic. In this regard, Fuchs is heavily influenced by gestalt theories and the idea of pattern formation when it comes to explaining action plans and actions. In short, during action, open loops between the environment and potential neural couplings are completed by resonance processes that link the environmental stimuli with planned action. In the case of a jazz solo, the improviser will combine planned moves and openness to the environment in order to properly resonate and act accordingly to make their solo.

Furthermore, following Fuchs’ use of the notion of “kinetic melodies” (which derives from Luria (1973) and has also been developed by Maxine Sheets-Johnstone (2011)), we can say that a jazz improvisor will deploy various learned motor gestalts and schemas to craft their particular improvisations. While there may be times when a musician decides to be more or less innovative and daring in their performance, any case of improvising will still fall back on at least some basic motoric components (e.g., a trumpet player must blow a certain way to create a sound and a pianist needs to shape their hands in certain ways to create chords). In a similar manner, following suggestions from Love (2017) ecological description of jazz improvisation, even highly skilled and creative jazz musicians, repeat runs and phrases with fair amounts of regularity during performance.

## Conclusion

Although we have contrasted representationalist and non-representationalist accounts of resonance, our intention was not to enter into the representation war (Clark, 2015), or to offer a full account of why one should be favored over the other. That would be a different project that has been subject to ongoing debate (see Downey, 2018; Williams, 2018). Rather, our aim has been to understand and explore the concept of resonance and its possible role in understanding the dynamical processes of brain-body-environment, and to highlight some problems and possible solutions in such an account. In this respect we’ve considered issues pertaining to isomorphism versus complimentarity, flexibility and agency, the weighting of system factors, and the possible role of environmental rearrangement or niche construction. These are issues that a dynamical resonance account needs to continue addressing as it develops.

One final addition to round out the solution is to appeal to the concept of *attunement* as either an addition or possible replacement to resonance. For instance, in considering the notion of resonance and its potential use in distributed cognition, Heft (2001) argues that:

terms such as…resonance are useful moves forward in helping us shed the dualistic trappings of inside/outside thinking. But at the same time, they may handicap thinking in a different way, by connotating a passive role for the individual relation…. Because knowing processes are marked by an individual selectively engaging the environment, a term with a more intentional connotation may better direct the thinking here. In this respect, *attunement* would seem to be more suitable (366, emphasis in original).

Since all of the previous theorists discussed are aware of this issue – especially if we include Gibson (1966) clarification that the brain is able to *self*-*tune*, and not merely reacting like a tuning fork excited by some soundwaves in the environment – it may be better to see attunement and resonance as co-extensive processes, rather than suggesting that we need to replace all uses of resonance with attunement or that the notion of self-tuning makes attunement somehow theoretically redundant.

There is likewise an important consideration regarding the different senses of passivity that could be at play. For instance, neither Fuchs nor Raja would argue that the brain, body, or world are static and unchanging. Indeed, even within the brain, and from single neurons all the way up to the whole brain, both acknowledge and embrace the fact that the constellation of neural activity evolves and develops over time, and this happens as it attunes to changing environments. The environment is likewise seen as integrally bound up with organisms instead of separate from them.

As such, we suggest that a full dynamical account of jazz performance will bring ecological resonance and enactive attunement to bear at the same time, where differing circumstances call for differing degrees of passivity and activity. At the same time, we hasten to add that it would be a mistake to think of the brain (or the organism, or the agent) as shifting between resonance and attunement. It is instead the case that the system is often in the process of doing both. The jazz soloist we discussed above is both resonating and attuning at the same time, after all, and we see no reason why that process would be different in other areas of cognition.

The full and dynamical account of our jazz improvisation case would therefore combine at least practices that involve resonance, attunement, and niche construction. This dynamical explanation offers a distinct story to tell regarding everything from why a musician played a specific note instead of another note, to how the entire ensemble can maintain their performance together over time.

Although novelty and uniqueness may be important for our everyday engagements, the general role of perception is not to come up with unique interpretations of the environment, but to properly orient us toward it. The same may be true for part of improvisation, at least to the extent that perception of what is already going on is needed while improvising. But such close perceptual orientation to the world does not seem to be true for all of improvisation. Likewise, while perception in jazz performance requires similar sorts of resonance as perception in other contexts – in order to perform, an improvising musician must be attuned and responsive to what has been played and is currently being played, just like someone walking down the street must be attuned and responsive to what is happening on the ground and around them – the jazz case goes beyond them as well. Jazz improvisers often place a premium on novelty and unique engagement with the environment, regardless of whether they stay close or move far away from source material during performance.

A continued engagement with this issue will require furthering considerations about resonance and attunement processes for agents acting in the world. We hope we have shown that we can approach such issues with a stronger sense of how ecological and enactive accounts of resonance resonate, and to what degree they hold in a fully developed account of music perception and production in particular, and perception and action in general.

## Author Contributions

KR and SG co-wrote and co-edited the entire document.

## Conflict of Interest

The authors declare that the research was conducted in the absence of any commercial or financial relationships that could be construed as a potential conflict of interest.

## Footnotes

1 Raja (2019) briefly considers and rejects ART as an acceptable gloss of Gibson’s notion of resonance because of its representational baggage.

2 While we are accepting that this move may be enough to distinguish Shepard’s account as representational, there have been arguments against the idea that structural representations are best understood as types of representations in the first place (cf. Segundo-Ortin and Hutto, 2019).

3 On one hand, one could say that Shepard also took a dynamical approach to cognition. On the other hand, insofar as representations are often offered as a stand-in for more dynamical kinds of explanations, we believe that the use of dynamical explanations here captures something importantly different from representationalists yet similar between enactivists and ecological psychologists.

4 Thanks to a reviewer for highlighting this more direct connection to Gibson.

5 We thank one of the journal reviewers for this reference.

6 The anticipation applies not only to the process of producing notes when the musician is playing, but to hearing what is produced - which means that the musician is not passively hearing, but actively listening, which is part of what resonant self-tuning implies. In this context, for the difference between hearing and listening, understood as intelligent and selective, see Roland Barthes (1985, 247) and for an enactive understanding of this, see Carvalho (2019).

7 Thanks to one of the reviewers for raising this point.

8 Nussbaum is clear that his account is representational but he does not explicitly consider it as a resonance-based account in the terms we have been exploring in this essay. However, considering the role of resonance for Shepard, we believe that this extrapolation is at least warranted as an analytical tool for our current purposes.

9 This is akin to the idea of habit breaking as found in improvised dance (see Kronsted and Gallagher, in press).

10 If, as we propose here, jazz performance involves quick, close to immediate, short-term resonance of the environment, rearranging furniture could support a form of slow, long-term (perhaps more metaphorical, although nonetheless material) resonance of the environment. Not only is it a rearrangement of affordances, but one could say of a furnished room that it “really resonates” (i.e., metaphorically resonates) with its inhabitant. In popular parlance, one might say that one “gets good vibes” from being in a particular environment.

11 One can argue that the reuse principle extends beyond neural plasticity to include “metaplasticity” (Malafouris, 2013), that is, the redeployment of artifacts, environmental resources, economic and cultural practices that, intentionally or unintentionally shape our cognitive and social practices (also see Gallagher, 2017).
